# The Targeted SMAC Mimetic SW IV-134 is a strong enhancer of standard chemotherapy in pancreatic cancer

**DOI:** 10.1186/s13046-016-0470-4

**Published:** 2017-01-17

**Authors:** Yassar M. Hashim, Suwanna Vangveravong, Narendra V. Sankpal, Pratibha S. Binder, Jingxia Liu, S. Peter Goedegebuure, Robert H. Mach, Dirk Spitzer, William G. Hawkins

**Affiliations:** 1 Department of Surgery, Barnes-Jewish Hospital and Washington University School of Medicine St. Louis, 660 S. Euclid Ave, Box 8109, Saint Louis, MO 63110 USA; 2 Department of Radiology, Washington University School of Medicine, St. Louis, MO USA; 3 Division of Gynecologic Oncology, Department of Obstetrics & Gynecology, Washington University School of Medicine, St Louis, MO USA; 4 Division of Public Health Sciences, Section of Oncologic Biostatistics, Washington University School of Medicine, St. Louis, MO USA; 5 Alvin J. Siteman Cancer Center, St. Louis, MO USA; 6 Department of Radiology, University of Pennsylvania, Philadelphia, PA USA; 7 Present Address: Department of Surgery, Cedars-Sinai Medical Center, 8700 Beverly Blvd, 8215-NT, Los Angeles, CA 90048 USA

**Keywords:** Sigma-2 receptors, Sigma-2/SMAC drug conjugate, Gemcitabine, Combination therapy, Pancreatic cancer

## Abstract

**Background:**

Pancreatic cancer is a lethal malignancy that frequently acquires resistance to conventional chemotherapies often associated with overexpression of inhibitors of apoptosis proteins (IAPs). We have recently described a novel means to deliver second mitochondria-derived activator of caspases (SMAC) mimetics selectively to cancer cells employing the sigma-2 ligand/receptor interaction. The intrinsic death pathway agonist SMAC offers an excellent opportunity to counteract the anti-apoptotic activity of IAPs. SMAC mimetics have been used to sensitize several cancer types to chemotherapeutic agents but cancer-selective delivery and appropriate cellular localization have not yet been considered. In our current study, we tested the ability of the sigma-2/SMAC drug conjugate SW IV-134 to sensitize pancreatic cancer cells to gemcitabine.

**Methods:**

Using the targeted SMAC mimetic SW IV-134, inhibition of the X-linked inhibitor of apoptosis proteins (XIAP) was induced pharmacologically and its impact on cell viability was studied alone and in combination with gemcitabine. Pathway analyses were performed by assessing caspase activation, PARP cleavage and membrane blebbing (Annexin-V), key components of apoptotic cell death. Single-agent treatment regimens were compared with combination therapy in a preclinical mouse model of pancreatic cancer.

**Results:**

The sensitizing effect of XIAP interference toward gemcitabine was confirmed via pharmacological intervention using our recently designed, targeted SMAC mimetic SW IV-134 across a wide range of commonly used pancreatic cancer cell lines at concentrations where the individual drugs showed only minimal activity. On a mechanistic level, we identified involvement of key components of the apoptosis machinery during cell death execution. Furthermore, combination therapy proved superior in decreasing the tumor burden and extending the lives of the animals in a preclinical mouse model of pancreatic cancer.

**Conclusion:**

We believe that the strong sensitizing capacity of SW IV-134 in combination with clinically relevant doses of gemcitabine represents a promising treatment option that warrants clinical evaluation.

**Electronic supplementary material:**

The online version of this article (doi:10.1186/s13046-016-0470-4) contains supplementary material, which is available to authorized users.

## Background

Pancreatic cancer is one of the most aggressive malignancies with an estimated 53,070 new cases and 41,780 deaths in the United States in 2016, with a 5-year survival rate of only 8% (Ref. [1]). Surgical intervention remains the only potential cure and even after surgery, the 5-year survival rate is only 10 - 25 percent due to the high rates of local recurrence and metastases [2]. Gemcitabine is a recommended treatment for pancreatic cancer and is used to treat both localized and metastatic disease. Gemcitabine can also be used in combination with radiation therapy [3]. Unfortunately, the benefits of gemcitabine therapy and other chemotherapies are rather limited. For example, gemcitabine chemotherapy only modestly improved overall survival to 6.8 months in patients with stage IV disease [4]. The response rate by Response Evaluation Criteria in Solid Tumors (RECIST criteria) for first line gemcitabine is only 9.4% [4]. A comparison between a combination of multiple active components such as FOLFIRINOX (5-FU, Irinotecan, Leucovorin and Oxaliplatin), *versus* a mix of Gemzar and albumin-complexed paclitaxel (Abraxane), did show similar disease control but both regimens were associated with substantial off-side toxicities [5]. Outcomes like these highlight the urgent need to develop more effective treatment options for patients with pancreatic cancer.

Pancreatic cancers use several mechanisms to evade apoptosis as they acquire resistance to conventional chemotherapy [6]. The inhibitor of apoptosis proteins (IAP) frequently contribute to drug resistance via blockade of caspase activation [7]. More specifically, the X-linked inhibitor of apoptosis proteins (XIAP) is a well-characterized member of the IAP family. It contains baculovirus IAP repeat (BIR) domains, of which BIR-2 is involved in blocking caspases-3/7 while BIR-3 interferes with activation of caspase-9 [8–10]. High intracellular XIAP levels have been attributed to chemoresistance in many pancreatic cancer cell lines as well as primary tumors [11]. Second mitochondria-derived activator of caspases (SMAC) is a mitochondrial protein that is released into the cytosol when cells are exposed to stress, and amplifies the apoptotic pathway by inhibiting IAP activity [12]. SMAC competitively binds to the caspase-binding domains of XIAP, resulting in their activation [12, 13]. Several SMAC mimetics have been described as potential therapeutics for cancer therapy and as sensitizers for traditional chemotherapeutics [14–17].

We have previously shown that sigma-2 receptors are overexpressed in human pancreatic cancer cells [18]. We have also demonstrated that sigma-2 ligands can enter and deliver additional drug cargos into pancreatic cancer cells [19]. We have recently described the novel drug conjugate SW IV-134, composed of the SMAC mimetic SW IV-52 and the sigma-2 ligand SW43 [20]. This potent cancer drug selectively targets the cancer cells via binding to the overexpressed sigma-2 receptor and induces cell death by delivering the SMAC mimetic SW IV-52 [20]. SW IV-134 has a high cytotoxic activity in the low micromolar range on pancreatic cancer cells in vitro and in mouse models of pancreatic cancer [20].

A key limitation of conventional chemotherapy is the toxicity to normal tissues due to a lack of selective cancer cell delivery. The purpose of this study was to evaluate the therapeutic potential of combining a targeted SMAC mimetic (SW IV-134) with gemcitabine in an effort to improve the efficacy of the non-targeted chemotherapeutic.

## Methods

### Compounds

The synthesis of SW IV-134 was performed in our laboratory and has been previously described [20]. Gemcitabine (Gemzar®) was purchased from Eli Lilly (Indianapolis, IN).

### Cell lines

PANC-1, CFPAC-1, BxPC-3, AsPC-1, and MIA PaCa-2 were obtained from American Type Culture Collection (ATCC, Manassas, VA). CFPAC-1 was cultured in Iscove’s modified medium with 4 mM L-glutamine, 1.5 g/L Sodium bicarbonate, and 10% fetal bovine serum (FBS). MIA PaCa-2 was cultured in Dulbecco's Modified Eagle's medium with 10% FBS and 2.5% horse serum. BxPC-3 and AsPC-1 were cultured in RPMI- 1640 medium with 10% FBS. Antibiotics, penicillin (100 μg/mL) and streptomycin (100 μg/mL) were added to the media. Cells were maintained in a humidified incubator at 37 °C with 5% CO_2._

### Evaluation of cytotoxicity in vitro

Cells were plated at a density of 1 × 10^4^/well in 96-well plates for 24 hours prior to treatment. SW IV-134 was dissolved in DMSO and diluted in culture medium to achieve a final concentration of 1 μM (the DMSO concentration was always kept below 1% and had thus no impact on the experimental results). Gemcitabine was dissolved in PBS to achieve a concentration of 0.5 μM. Cells were treated with the SW IV-134, gemcitabine, and combination of both drugs that contained 1 μM of SW IV-134 and 0.5 μM of gemcitabine. Cell viability was determined 3 - 4 days after treatment using CellTiter-Glo Luminescent Viability Assay (Promega, Madison, WI). Luminescence signal was measured using a multi-mode microplate reader (Bio-Tek, Winooski, VT). All assays were performed in triplicates.

### Lentivirus mediated XIAP knockdown

The lentiviral constructs, pLKO.1 for XIAP (sh-1, TRCN0000003785; sh-2, TRCN0000003787) and Luciferase (sh-Luc) were obtained from Washington University genome center. shRNA constructs were transfected into HEK293T cells together with the lentiviral packaging plasmids VSVG (envelope) and ∆8.9 (gag, pol), using Fugene 6 (Roche, Indianapolis, IN). Viral supernatants were collected at 48 and 72 hours and added to PANC-1 and MIA PaCa-2 cells in the presence of protamine sulfate (10 μg/mL). Infected cells were selected with Puromycin (2 μg/mL). The drug selection process was monitored via GFP fluorescence (as part of the lentiviral vectors) and was in the range of 90% positive cells.

### Immunoblotting

Cells were lysed in radioimmunoprecipitation assay buffer [50 mM Tris, 150 mM sodium chloride, 1 mM EDTA, 1% Nonidet P40, and 0.25% SDS (pH 7.0)]. The buffer was supplemented with complete protease inhibitor cocktail (Roche, Mannheim, Germany). Protein concentration was measured by BCA protein assay kit (Thermo Fisher Scientific, Rockford, IL). Samples containing equal amounts of protein were run on NuPAGE Bis-Tris 4 - 12% gradient gels and then transferred onto PVDF membranes (Life Technologies, Grand Island, NY). The membranes were incubated in blocking buffer (5% dry milk) for 1 hour, followed by addition of the respective primary antibodies at 4 °C overnight. The following day, membranes were washed with TBS-T and incubated with HRP-conjugated secondary antibodies at room temperature for 1 hour. SuperSignal West Dura Substrate (Thermo Fisher Scientific Rockford, IL) was used to detect the secondary antibodies. Primary and secondary antibodies for capase-3, cleaved caspase-3, Poly ADP-ribose polymerases (PARP), cleaved PARP, and XIAP were purchased from Cell Signaling Technology (Danvers, MA). Primary and secondary actin antibodies were purchased from Santa Cruz (Dallas, TX). Antibody dilutions were made according to the manufacturer’s instructions.

### In vitro evaluation of apoptosis (Annexin-V staining)

AsPC-1 cells were seeded in 6-well plates at a density of 5 × 10^5^/well for 24 hours. Cells were treated for 48 hours with 0.5 μM of SW IV-134, 0.5 μM of gemcitabine, equimolar concentration of both drugs, and DMSO as a control. Apoptosis was detected using Annexin-V FITC Kit (Biolegend, San Diego, CA). Propidium iodide was added to differentiate early apoptotic cells from necrotic and late stage apoptotic cells. Cells were prepared according to the manufacturer's instructions and analyzed with a FACSCalibur flow cytometer (BD Biosciences, San Jose, CA).

### In vitro caspase activation assays

Caspase-3, 8 and 9 activities were measured in PANC-1 and AsPC-1 cells using Caspase-Glo Assay Systems (Promega, Madison, WI) according to the manufacturer’s instructions. This assay is based on luminogenic caspase substrates which are cleaved by activated caspases resulting in generation of a luminescence signal. Cells were seeded at a density of 1 × 10^4^ in 96-well plates for 24 hours. Then, they were treated for 48 hours with 1 μM of SW IV-134, 0.5 μM of gemcitabine, combination of the two drugs, and DMSO as a control. The contents of the plate were mixed using an orbital shaker for 30 seconds and incubated at room temperature for 90 minutes. Luminescence signal was measured using a multi-mode microplate reader (Bio-Tek, Winooski, VT).

### In vivo assessment of tumor growth, survival, and toxicity

Immunocompromised female nude mice (6 weeks old, Harlan Laboratories, Indianapolis, IN) were injected in the right flank with 200 μL single cell suspension of 1 × 10^6^ AsPC-1 cells in RPMI medium. Treatment started when tumors reached approximately 5 mm in diameter. Mice were randomized into four groups (*n* = 14). The groups received daily i.p. injections with 100 μL of vehicle (25% cremophor in H_2_O), weekly gemcitabine (3 mg), daily SW IV-134 (750 nmoles) with and without weekly gemcitabine (3 mg). Tumors were measured every other day with a digital caliper and the volumes were calculated using the equation V = d_1_ (d2)^2^/2, (V = volume, d_1_ = larger diameter, d_2_ = smaller diameter). Following conclusion of treatment at day 21, five mice from each group were sent to the Division of Comparative Medicine at our institution for toxicity evaluation. Blood was collected for complete blood count (CBC) and biochemical analysis (AST, ALT, BUN, total bilirubin, and Cr). Organs were examined grossly and histologically. Mice were euthanized when tumors reached a diameter of 2 cm or ulcerated. Animal euthanasia was performed using a carbon-dioxide (CO_2_) chamber. Mice were placed in the CO_2_ chamber (≤10 mice at a time) and 100% CO_2_ was introduced at a slow rate, replacing 30% of the chamber volume in 1 minute. Mice were exposed to CO_2_ for five minutes followed by a two minute dwell period. Animal studies were carried out in accordance with the Washington University Division of Comparative Medicine guidelines for care and use of laboratory animals. The protocol was approved by the Animal Studies Committee of Washington University (protocol 20130073).

### Statistics

Statistical analyses and data plotting were performed using GraphPad Prism software version 5 (San Diego, CA). Results were expressed as mean ± SEM of at least 3 biological replicates. One-way ANOVA was used to analyze the differences in viability and caspase activity assays. Unpaired two tailed t-tests were used to evaluate the difference in CBC, biochemistry analyses, viability of knock-down cells, and to confirm the difference in subgroups. Two-way ANOVA was used to analyze the difference in tumor sizes. Kaplan-Meier survival analysis was used and the difference between the groups was compared with a log-rank test. A *p-*value < 0.05 was considered significant for all analyses.

## Results

### Low XIAP expression sensitizes pancreatic cancer cells to gemcitabine

**Fig. 1:**
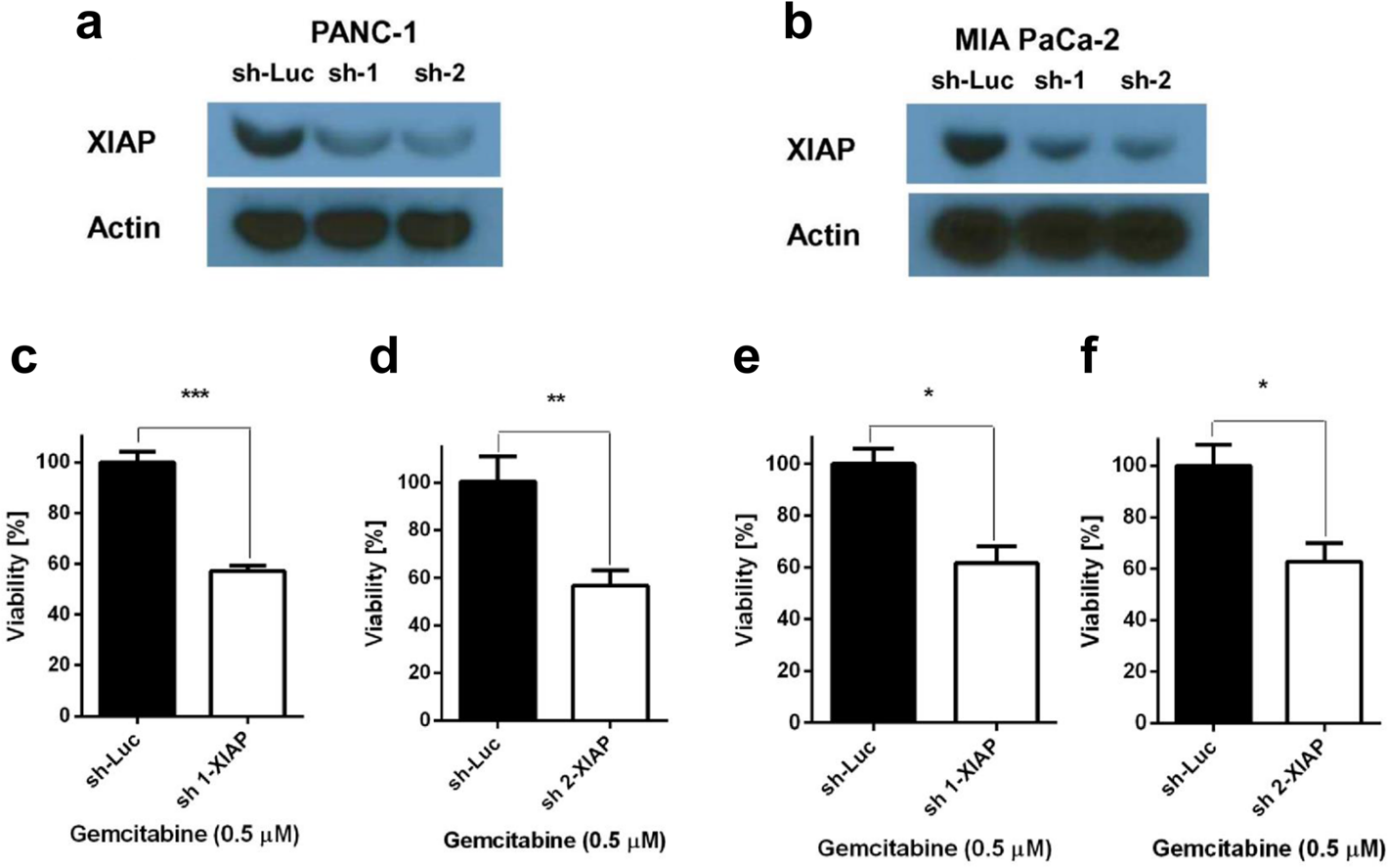
Down regulation of XIAP expression sensitizes pancreatic cancer cells to gemcitabine. **a** PANC-1 and **b** MIA PaCa-2 cells were infected with lentiviral vectors encoding two XIAP-specific shRNAs (sh-1 and sh-2) and a luciferase-specific control (sh-Luc). Stable cell pools were generated using puromycin drug selection. XIAP knockdown was confirmed by Western blot analysis and resulted in expression levels of 49% - 37% (PANC-1, sh-1, sh-2) and 42% - 28% (MIA PaCa-2, sh-1, sh-2), respectively, with the sh-2 clone being slightly more potent in both cell lines. The cells were treated with low-dose gemcitabine (0.5 μM) and the viability was assessed 4 days later. While treatment of the sh-Luc controls did not induce cell death, the percentage of live cells after treatment with gemcitabine decreased by ~40% for both cell lines and both XIAP shRNA clones. **c** and **d,** PANC-1, ***p < 0.001 and **p < 0.01. **e** and **f,** MIA PaCa-2, *p < 0.05 (*n* = 3)

XIAP has been shown to be involved in resistance of pancreatic cancer to conventional chemotherapy [11, 21]. In order to compensate the anti-apoptotic properties of this molecule, much higher doses of, e.g. gemcitabine might be required to achieve an adequate treatment benefit. High doses of chemotherapy are almost inevitably correlated with substantial side effects for the patients. In order to verify that low XIAP expression increases the sensitivity of pancreatic cancer cells to gemcitabine, we genetically decreased the expression level of XIAP using shRNA. The pancreatic cancer cell lines PANC-1 and MIA PaCa-2 were stably infected with two lentiviral shRNA clones directed against human XIAP. Cells infected with luciferase-specific shRNA were used as controls. Western blot analysis confirmed reduced XIAP expression levels for both cell lines (Fig. 1a and b). To assess the impact of reduced XIAP expression levels on gemcitabine sensitivity, the cells were treated with gemcitabine for 4 days. While the controls did not show any signs of cell death induction, the viability of XIAP-depleted cells was reduced to ~60% (Fig. 1c, d, e and f). Of note, we have previously shown the effect of SW IV-134 on functional blockage of XIAP and rapid degradation of cIAP-1/2, which leads to NIK-dependent TNFα production and augmented target cell apoptosis [20, 22]. These data confirm published reports about the important role of XIAP in the chemoresistance of pancreatic cancer cells to gemcitabine and validate the experimental model used to assess our tumor-targeted SMAC mimetics.

### SW IV-134 sensitizes pancreatic cancer to gemcitabine

**Fig. 2:**
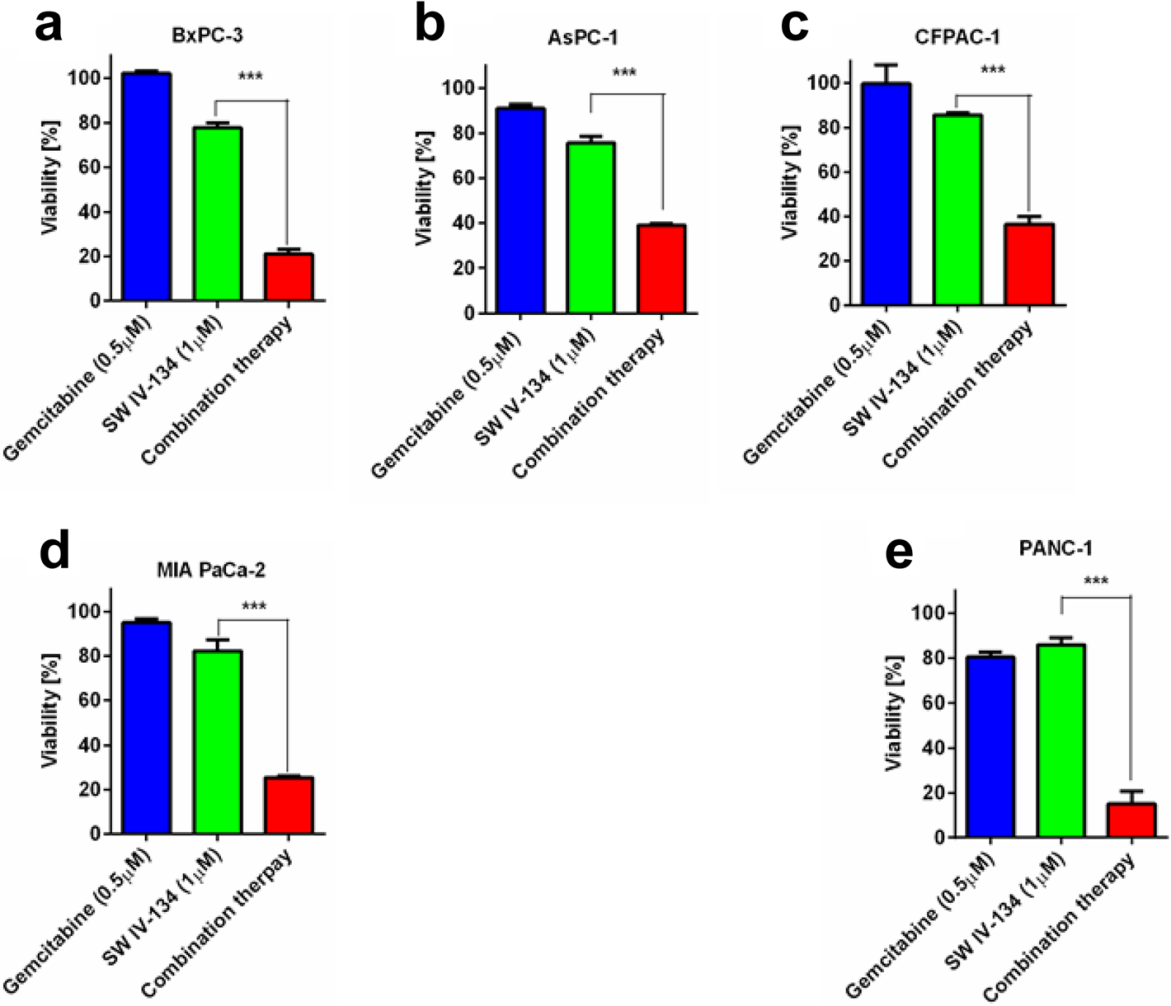
SW IV-134 sensitizes pancreatic cancer cells to gemcitabine. **a** BxPC-3, **b** AsPC-1, **c** CFPAC-1, **d** MIA PaCa-2, and **e** PANC-1 cells were treated with SW IV-134 (1 μM), gemcitabine (0.5 μM), or in combination with the two drugs using the same concentrations. Titer-Glo viability assays were performed after 3 - 4 days post treatment (MIA PaCa-2, 4 days; all other cell lines, 3 days). The data were normalized to DMSO treated control cells (***p < 0.001) (*n* = 3)

Since the SMAC moiety of our cancer-targeted small molecule drug conjugate SW IV-134 displaces XIAP from its designated caspases (3/7 and 9), leading to their activation [23, 24], we hypothesized that SW IV-134 would also improve the sensitivity of pancreatic cancer cells to gemcitabine, known to develop resistance against this chemotherapeutic [25, 26]. Pancreatic cancer cells were thus treated with our targeted SMAC mimetic to explore if the cells could be rendered sensitive to gemcitabine pharmacologically. We chose to treat all cell lines with sublethal concentrations of the respective drugs. For example, the IC_50_ data for SW IV-134 were already established in our previous report and ranged from 6.3 to 9.2 μM [20]. We used this information as a guide and selected a 1 μM concentration of SW IV-134 for all experiments. The sublethal concentration for gemcitabine was identified in a pilot study (see also Fig. 1c, d, e and f). At the selected doses, single-agent treatment with SW IV-134 or gemcitabine showed minimal reduction in cell viability (Fig. 2, 73% - 85% and 80% - 100%, respectively). In contrast, the combination of the two drugs substantially decreased the cell viability to as low as ~15% (Fig. 2e, *p* < 0.001 for all analyses). The effects of the drugs, when used in combination, were much greater than the sum of the individual reagents, suggesting a synergistic effect (Fig. 2).

### SW IV-134 augments gemcitabine-induced activation of both the intrinsic and the extrinsic pathways of apoptosis

**Fig. 3:**
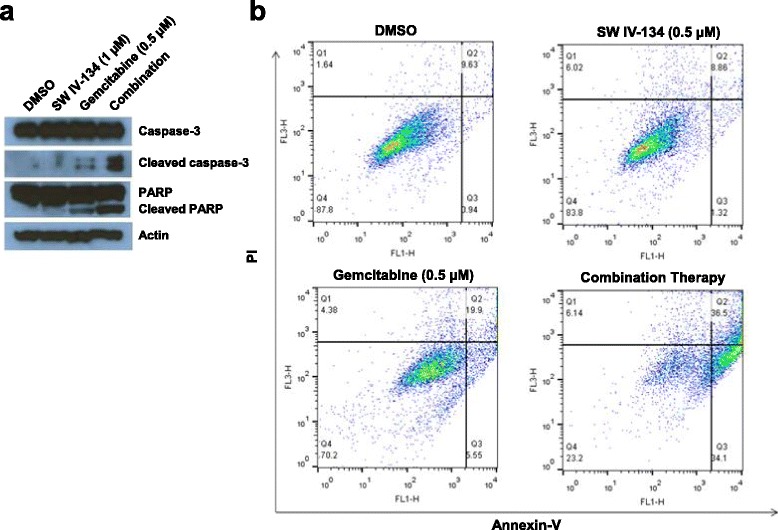
SW IV-134 augments gemcitabine-induce apoptotic cell death. **a** PANC-1 cells were treated with DMSO (control), SW IV -134 (1 μM), gemcitabine (0.5 μM), and a combination of the two drugs at their respective concentrations. Apoptotic cell death was assessed by Western blot analysis via monitoring the activation status of caspase-3 and PARP 48 hours after treatment. Beta-Actin was used a loading control. **b** A complementary flow cytometry-based assay was employed to assess apoptotic cell death via Annexin-V/PI staining. AsPC-1 cells were treated essentially as in (**a**) but SW IV-134 was (0.5 μM). After two days, the cells were submitted to Annexin-V/PI staining. Shown are representative FACS dot plots for the indicated treatment conditions (*n* = 4). DMSO-treated cells served as a control. The Annexin-V+/PI- cells in the lower right quadrant represent early apoptotic cells

Both SW IV-134 and gemcitabine are capable of inducing cell death via apoptosis [20, 27]. Since combination of the two compounds correlated with enhanced killing profiles using sublethal concentrations of the individual components, we asked if these effects were a consequence of augmented apoptosis induction. Cleaved caspase-3 (the major executioner caspase of apoptosis) and cleaved PARP (a major sensor of DNA damage and cell stress) were used to monitor pathway induction via Western blotting [28, 29]. PANC-1 cells were treated in vitro with gemcitabine, in the absence or presence of SW IV-134. Following treatment, cell lysates were prepared and submitted to Western blot analysis. Low-dose SW IV-134 alone was unable to produce either activated caspase-3 or PARP, while low-dose gemcitabine resulted in limited generation of the two apoptosis indicators. Only the two drugs combined produced a substantial increase in signal intensities for the activated apoptosis markers caspase-3 and PARP, respectively (Fig. 3a).

For further assessment of apoptosis involvement during combination treatment, AsPC-1 cells were exposed to SW IV-134 and gemcitabine alone, an equimolar mix of the drugs and DMSO (control). The cells were subsequently assessed for membrane blebbing (Annexin-V) and PI uptake. The combination of both drugs resulted in the highest degree of early apoptosis with 34% of cells being Annexin-V+/PI– (Fig. 3b). Only gemcitabine single-agent treatment resulted in a somewhat elevated level of early apoptosis (5.5%), while none of the other groups showed clear evidence of treatment effects (DMSO and SW IV-134 with 0.9% and 1.3%, respectively). These data further suggest that low-dose combination therapy mediates strong cytotoxicity in an apoptosis-dependent fashion.

**Fig. 4:**
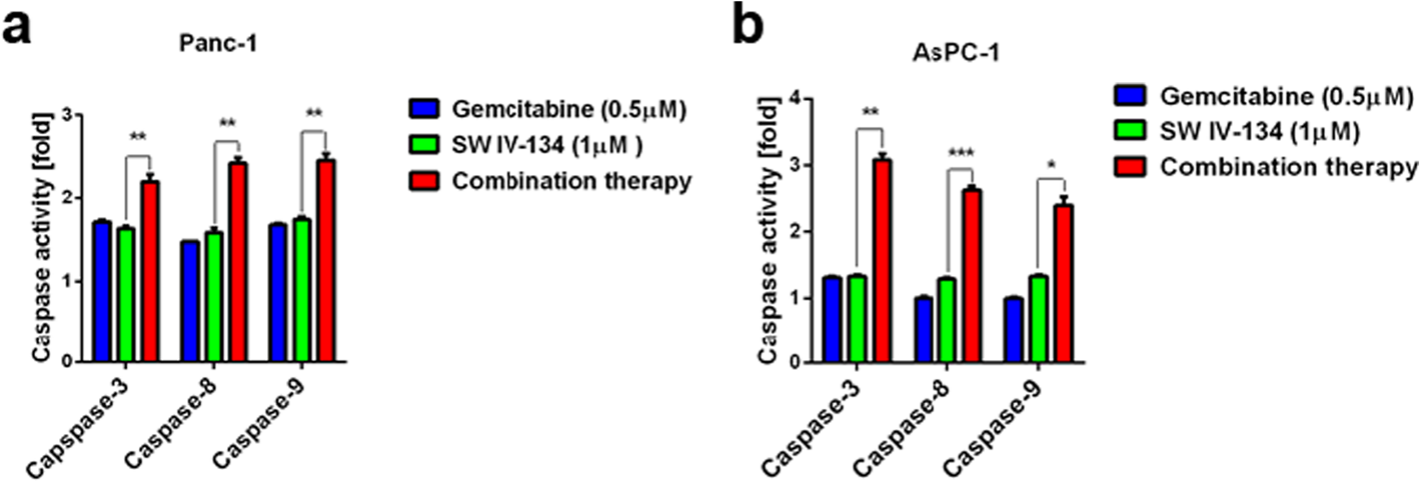
SW IV-134 induces both intrinsic and extrinsic pathways of apoptosis. **a** PANC-1 and **b** AsPC-1 cells were treated with SW IV-134 (1 μM), gemcitabine (0.5 μM), and a combination of the two drugs at their respective concentrations. The activation status of caspases 3, 8 and 9 were measured using a Caspase-Glo Assay System (see Methods for details). The data are normalized to the luminescence signals for each caspase on cells treated with DMSO (baseline). (*n* = 3; **p < 0.01)

Endogenous SMAC competitively displaces XIAP from the BIR-3 binding groove that mediates caspase-9 interaction, leading to its activation [8]. We have previously shown that SW IV-134 is capable of inducing caspase-9 cleavage (intrinsic death pathway), which mimics the activity of the native SMAC molecule [20]. SW IV-134 also induces NF-κB-mediated, TNFα-dependent extrinsic pathway activation via degradation of cellular inhibitor of apoptosis proteins-1 (cIAP-1) [20, 22]. Gemcitabine has been shown to activate caspases 8 and 9 [27]. To evaluate the relative contribution of these two pathways in apoptosis following combination treatment, the activation status of caspases 3, 8, and 9 was monitored in PANC-1 and AsPC-1 cells. In PANC-1, treatment with the individual drugs increased all caspase levels ~1.7 - fold above baseline, while the combination of both drugs led to a ~2.4 - fold increase (+41%, Fig. 4a, *p* < 0.01). While treatment of AsPC-1cells with individual drugs increased all caspase levels ~1.3-fold above baseline, the combination of both drugs relative to SW IV-134 treatment led to an ~3-fold increase in caspase-3 activity, the executioner caspase of programmed cell death (+130%, Fig. 4b, *p* < 0.01). These results are consistent with our cytotoxicity data and suggest that both pathways of apoptosis are activated much more efficiently when gemcitabine is combined with SW IV-134.

### SW IV-134 enhances gemcitabine therapy in a preclinical xenograft model of pancreatic cancer

**Fig. 5:**
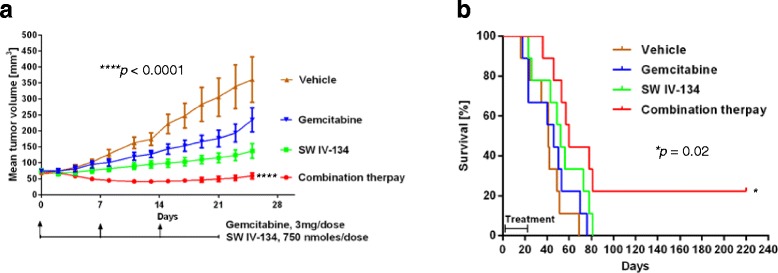
SW IV-134 augments gemcitabine in a mouse xenograft model of pancreatic cancer and improves survival. **a** Immunocompromised female nude mice were injected in the right flank with 200 μL single cell suspension of 1 × 10^6^ AsPC-1 cells (*n* = 9). Treatment started when tumors reached ~5 mm in diameter. The mice received the following treatments by i.p. injection: SW IV-134 alone (daily 750 nmoles/100 μL), vehicle (daily 100 μL as control), gemcitabine (weekly 3 mg/100 μL), and a combination of SW IV-134 (daily 750 nmoles/100 μL) and gemcitabine (weekly 3 mg/100 μL). The tumors were measured every other day using digital calipers. Of note, at the end of the treatment interval (21 days), the tumors of the mice receiving combination therapy were significantly smaller than the tumors of the other groups (*p* < 0.0001). **b** Kaplan-Meier survival curve of mice in (**a**). The median survival of mice treated with combination therapy was 60 days as compared to 52, 41 and 46 days for the SW IV-134, vehicle and gemcitabine groups, respectively (*p* = 0.02)

In an attempt to verify our in vitro findings in an appropriate animal model of pancreatic cancer, we inoculated AsPC-1 cells into the flanks of female nude mice (xenograft model). When the tumors reached a diameter of ~5 mm, the mice were treated with gemcitabine alone, SW IV-134 alone, or a combination of the two cancer drugs. Mice treated with vehicle served as the control group. While the individual drugs slowed the tumor growth to a small degree (SW IV-134 > gemcitabine), combination therapy was substantially more effective (Fig. 5a, *p <* 0.0001). The median survival of mice treated with combination therapy was 60 days as compared to 41, 46 and 52 days for the vehicle, gemcitabine and SW IV-134 single-agent treatment groups, respectively (Fig. 5b, *p* = 0.02). Two out of 9 mice (22%) receiving combination therapy survived for more than 7 months, while no long-term survivors were observed in all of the other groups. Long-term survivors are highly unusual in xenograft models of pancreatic cancer. With the exception of anticipated decreases in WBC counts due to gemcitabine-associated side effects, we did not detect any other significant differences in laboratory parameters compared to the control group (Additional file 1: Table S1 and Additional file 2: Table S2). Apart from mild peritonitis at the site of the injection, organ analyses (brain, heart, lungs, alimentary tract, kidneys, liver and pancreas) did not reveal signs of adverse drug effects. We observed no abnormalities in mouse behavior (failure to groom or weight loss) and no drug related deaths in our experiments.

## Discussion

Pancreatic cancer has a poor prognosis and novel therapeutic approaches are desperately needed. Gemcitabine has been combined safely with several other chemotherapeutics in efforts to improve outcomes but success has been very modest. For example, combination of gemcitabine with erlotinib or capecitabine mildly improved overall survival by a few weeks [30, 31]. Recently, an intensive multi-drug combination has shown more significant gains in survival. The regimen includes doses of 5-FU, oxaliplatin, irinotecan, and leucovorin (FOLFIRINOX). Overall survival improved from 6.8 months to 11.1 months when gemcitabine was added but at the cost of significant toxicity. FOLFIRINOX was associated with substantially more adverse effects, including febrile neutropenia, thrombocytopenia, diarrhea, and sensory neuropathy [4]. As a consequence, this treatment option is only suitable for the healthiest of patients suffering from pancreatic cancer.

Evading apoptosis is an important mechanism of acquired or preexisting chemoresistance in pancreatic cancer [32, 33]. Restoring the ability to undergo programmed cell death is therefore an attractive strategy to enhance treatment efficiencies [34]. IAPs belong to a family of proteins frequently involved in resistance of pancreatic cancer to chemotherapeutics [28, 35]. XIAP is the most prominent and potent member of this family and its transcriptional down regulation or pharmacologic blockade using SMAC mimetics has been shown to sensitize pancreatic cancer cells to gemcitabine [11, 28]. Interestingly, depletion of cIAP-1 and cIAP-2 alone was not sufficient to sensitize pancreatic cancer cells to gemcitabine [17]. In agreement to these earlier studies, we confirmed that decreased XIAP levels substantially improved sensitivity to gemcitabine, even in the context of residual XIAP expression (Fig. 1). We therefore believe that this up-regulated cellular survival pathway represents an attractive target in patients with pancreatic cancer. In order to exploit XIAP as a putative pancreatic cancer target most efficiently, delivery of its antagonist, the SMAC peptidomimetic, needs to be rendered cancer selective, since a non-selective mimetic would increase the risks for systemic toxicities. By linking a SMAC mimetic to the sigma-2 ligand SV119, we created a targeted therapeutic capable of delivering its payload directly into the cancer cells [20, 22]. It is important to note that SMAC mimetics, including our targeted SW IV-134, induce cancer cell death via complex mechanisms, currently only incompletely elucidated. While XIAP is functionally blocked, cIAP-1/2 is rapidly degraded, which leads to NIK-dependent TNFα production and augmented target cell killing by inducing apoptosis [8, 20, 22]. Even though SW IV-134 targets the same pathways as SMAC itself, we found it to be far more effective than the unconjugated SMAC mimetic [20]. The reason(s) for its enhanced activity profile is not completely understood and constitutes an active research area but we believe it is likely caused by an enhanced and cancer-selective uptake/internalization mechanism, as we have recently shown for the targeted delivery of an erastin analog to treat pancreatic cancer in vitro and in vivo [36].

This is the first description of using SW IV-134 in combination with a standard chemotherapeutic, gemcitabine, in PDAC. However, it might achieve even better treatment outcomes when combined with non-standard experimental therapies, such as targeted TRAIL biologics [37]. In order to show the potential synergy between SW IV-134 and gemcitabine, we reduced the doses of the individual drugs. We are currently in the process of designing preclinical models of PDAC in order to identify the maximum tolerated dose in vivo. At the reduced doses, combination of SW IV-134 with gemcitabine significantly slowed tumor growth and increased in the median overall survival of our animals. Apart from a decrease in the WBC count, commonly seen following gemcitabine chemotherapy [38], and mild peritonitis at the site of injection, no toxicities were observed.

Sigma-2 receptors are up-regulated in many cancer types [39, 40] and SMAC mimetics have been shown to sensitize many types of cancer to a wide array of chemotherapeutic agents [17, 41, 42]. As a result, we believe that SW IV-134 could potentially be used to sensitize several additional sigma-2-expressing malignancies, including head and neck tumors, breast cancer, lymphomas [43], and ovarian cancer [22] to different chemotherapeutics. As such, the combination therapy approach employed here may have implications beyond the treatment of patients with pancreatic adenocarcinoma.

## Conclusions

In this study, we described the ability of the sigma-2 receptor-targeted SMAC mimetic SW IV-134 to act as a sensitizing agent for chemotherapy in a model of pancreatic cancer. This approach merits further investigation, since this cancer has been recalcitrant to most standard therapies. This work is significant because sigma-2 receptors are highly expressed in many types of cancer. As a result, we believe that the targeted combination strategy will likely work for additional malignancies. Since many chemotherapeutics, including SW IV-134, activate the intrinsic arm of the apoptotic pathway, it may work well to accentuate the effects of a variety of therapeutics that trigger complementing effector arms of programmed cell death, such as the TRAIL-induced extrinsic death pathway.

## Additional files

Additional file 1: Table S1. Combined SW IV-134 and gemcitabine therapy induces a moderate decrease in WBC count and has no effect on the other CBC parameters in tumor-bearing mice. Complete blood count (CBC) of immunocompromised nude mice treated daily with SW IV-134 and weekly gemcitabine for 3 weeks compared to vehicle (control). WBC is reduced in the drug group, with mean = 2.4 compared to 4.7 in the control group (*p* = 0.02). The differences in the rest of cell counts between the two groups were not statistically significant. (PDF 75 kb)
Additional file 2: Table S2. Combined SW IV-134 and gemcitabine therapy does not induce changes in serum chemistries in tumor-bearing mice. Biochemical analysis of immunocompromised nude mice treated daily with SW IV-134 and weekly gemcitabine for 3 weeks compared to vehicle (control). The differences in laboratory values between the two groups were not statistically significant. (PDF 16 kb)

## Abbreviations

ALT: Alanine aminotransferase
AST: Aspartate aminotransferase
BUN: Blood urea nitrogen
cIAP-1: Cellular inhibitor of apoptosis proteins-1
Cr: Creatinine
DMSO: Dimethyl sulfoxide
i.p.: intraperitoneal
IAP: Inhibitor of apoptosis proteins
PARP: Poly ADP-ribose polymerase
RECIST: Response Evaluation Criteria in Solid Tumors
SMAC: Second mitochondria-derived activator of caspases
XIAP: X-linked inhibitor of apoptosis proteins
