# Supplementary material for: The Targeted SMAC Mimetic SW IV-134 is a strong enhancer of standard chemotherapy in pancreatic cancer
Source: J Exp Clin Cancer Res. 2017 Jan 17;36:14. doi: 10.1186/s13046-016-0470-4 (PMC5240213; doi:10.1186/s13046-016-0470-4)
Supplement: Additional file 1: Table S1. — Combined SW IV-134 and gemcitabine therapy induces a moderate decrease in WBC count and has no effect on the other CBC parameters in tumor-bearing mice. Complete blood count (CBC) of immunocompromised nude mice treated daily with SW IV-134 and weekly gemcitabine for 3 weeks compared to vehicle (control). WBC is reduced in the drug group, with mean = 2.4 compared to 4.7 in the control group (p = 0.02). The differences in the rest of cell counts between the two groups were not statistically significant. (PDF 75 kb) [file 13046_2016_470_MOESM1_ESM.pdf]

Additional Table S1

Mice	WBC ($10^3/\mu L$)	RBC ($10^6/\mu L$)	HGB (g/dl)	PCV (%)	MCV (u)	MCH (pg)	MCHC (%)	Platelets ($10^3/\mu L$)
**Drug 1**	3.48	8.68	12.6	44.0	50.7	14.5	28.6	852
**Drug 2**	3.52	7.64	11.2	37.2	48.7	14.7	30.1	820
**Drug 3**	1.30	8.07	12.1	38.1	47.2	15.0	31.8	610
**Drug 4**	2.50	7.46	10.9	36.6	49.0	14.6	28.8	767
**Drug 5**	1.02	7.60	12.0	38.0	50.0	15.8	31.6	620
**Control 1**	4.72	8.63	13.2	43.3	50.2	15.3	30.5	1107
**Control 2**	2.76	7.07	10.5	35.6	50.4	14.9	29.5	720
**Control 3**	4.14	8.57	13.0	43.6	50.9	15.2	29.8	677
**Control 4**	5.38	8.56	13.2	44.6	52.1	15.4	29.6	771
**Control 5**	6.72	8.94	13.2	43.8	49.0	14.8	30.1	1673

Table S1. Combined SW IV-134 and gemcitabine therapy induces a moderate decrease in WBC count and has no effect on the other CBC parameters in A thymic nude mice. CBC of Athymic Nude mice treated daily with SW IV-134 and weekly gemcitabine for 3 weeks compared to vehicle (control). WBC is reduced in the drug group, with mean= 2.4 compared to 4.7 in the control group p = 0.02. The differences in the rest of cell counts between the 2 groups are not statistically significant.
