# Supplementary material for: The Targeted SMAC Mimetic SW IV-134 is a strong enhancer of standard chemotherapy in pancreatic cancer
Source: J Exp Clin Cancer Res. 2017 Jan 17;36:14. doi: 10.1186/s13046-016-0470-4 (PMC5240213; doi:10.1186/s13046-016-0470-4)
Supplement: Additional file 2: Table S2. — Combined SW IV-134 and gemcitabine therapy does not induce changes in serum chemistries in tumor-bearing mice. Biochemical analysis of immunocompromised nude mice treated daily with SW IV-134 and weekly gemcitabine for 3 weeks compared to vehicle (control). The differences in laboratory values between the two groups were not statistically significant. (PDF 16 kb) [file 13046_2016_470_MOESM2_ESM.pdf]

Additional Table S2

Mice	BUN (mg/dL)	Creatinine (mg/dL)	ALT (u/L)	AST (u/L)	T. Bili (mg/dL)	Glucose (mg/dL)	Total Protein (g/dL)
Drug 1	41	0.36	68	52	0.10	181	5.7
Drug 2	41	0.33	63	77	0.06	114	4.8
Drug 3	38	0.32	84	121	0.07	219	5.4
Drug 4	39	0.27	54	62	0.17	140	5.6
Drug 5	35	0.26	52	72	0.11	153	5.5
Control 1	46	0.37	69	61	0.13	162	5.5
Control 2	35	0.33	57	58	0.12	170	5.3
Control 3	34	0.37	59	55	0.13	195	5.8
Control 4	33	0.27	62	189	0.08	168	5.3
Control 5	28	0.27	48	54	0.14	221	5.5

Table S2. Combined SW IV-134 and gemcitabine therapy does not induce changes in serum chemistries in tumor-bearing mice. Biochemical analysis of CBC of Athymic Nude mice treated daily with SW IV-134 and weekly gemcitabine for 3 weeks compared to vehicle (control). The differences in laboratory values between the 2 groups are not statistically significant.
